# [^125^ I]IodoDPA-713 Binding to 18 kDa Translocator Protein (TSPO) in a Mouse Model of Intracerebral Hemorrhage: Implications for Neuroimaging

**DOI:** 10.3389/fnins.2018.00066

**Published:** 2018-02-22

**Authors:** Frederick Bonsack, Catherine A. Foss, Ali S. Arbab, Cargill H. Alleyne, Martin G. Pomper, Sangeetha Sukumari-Ramesh

**Affiliations:** ^1^Department of Neurosurgery, Medical College of Georgia, Augusta University, Augusta, GA, United States; ^2^Russell H. Morgan Department of Radiology and Radiological Science, Johns Hopkins School of Medicine, Johns Hopkins University, Baltimore, MD, United States; ^3^Laboratory of Tumor Angiogenesis, Georgia Cancer Center, Department of Biochemistry and Molecular Biology, Augusta University, Augusta, GA, United States

**Keywords:** [^125^ I]IodoDPA-713, microglial activation, stroke, intracerebral hemorrhage, gliosis

## Abstract

Intracerebral hemorrhage (ICH) is a fatal stroke subtype with significant public health impact. Although neuroinflammation is a leading cause of neurological deficits after ICH, no imaging tool is currently available to monitor brain inflammation in ICH patients. Given the role of TSPO in neuroinflammation, herein we investigate whether a second-generation TSPO ligand, [^125^ I]IodoDPA-713 can be used to monitor the changes in TSPO expression in a preclinical model of intracerebral hemorrhage. Male CD1 mice were subjected to ICH/Sham. The brain sections, collected at different time points were incubated with [^125^ I]IodoDPA-713 and the brain uptake of [^125^ I]IodoDPA-713 was estimated using autoradiography. The specificity of [^125^ I]IodoDPA-713 binding was confirmed by a competitive displacement study with an unlabeled TSPO ligand, PK11195. [^125^ I]IodoDPA-713 binding was higher in the ipsilateral striatum with an enhanced binding observed in the peri-hematomal brain region after ICH, whereas the brain sections from sham as well as contralateral brain areas of ICH exhibited marginal binding of [^125^ I]IodoDPA-713. PK11195 completely reversed the [^125^ I] IodoDPA-713 binding to brain sections suggesting a specific TSPO-dependent binding of [^125^ I]IodoDPA-713 after ICH. This was further confirmed with immunohistochemistry analysis of adjacent sections, which revealed a remarkable expression of TSPO in the areas of high [^125^ I]IodoDPA-713 binding after ICH. The specific as well as enhanced binding of [^125^ I]IodoDPA-713 to the ipsilateral brain areas after ICH as assessed by autoradiography analysis provides a strong rationale for testing the applicability of [^125^ I]IodoDPA-713 for non-invasive neuroimaging in preclinical models of ICH.

## Introduction

Intracerebral hemorrhage (ICH) is a detrimental subtype of stroke caused by bleeding within the brain tissue itself. ICH accounts for 10–20% of strokes and has a fatality rate of 40 and 54% at 30 days and 1 year, respectively (An et al., 2017). Notably, there is no substantial change in fatality rate over the last 40 years (An et al., 2017). Moreover, the survivors of ICH often exhibit neurological deficits partly because of the secondary brain insults caused by the released blood components in the brain parenchyma (Elliott and Smith, 2010; Babu et al., 2012).

The pathophysiology of ICH includes both primary as well as secondary brain damage. The primary damage, occurring within minutes to hours after the initial brain hemorrhage is mostly caused by the mass effect of the hematoma. In contrast, the secondary brain damage develops from hours to days after the initial brain insult and can lead to severe neurological disability(Aronowski and Zhao, 2011; Belur et al., 2013). The secondary brain damage is mainly attributed to the inflammatory and oxidative responses to released blood components and associated neurotoxicity (Aronowski and Zhao, 2011; Belur et al., 2013). Neuroinflammation, often characterized by microglial activation, plays a critical role in the pathophysiology of ICH, and the brain inflammatory response correlates with the expansion of hematoma, neurodegeneration, and poor functional outcomes (Platt et al., 1998; Hickenbottom et al., 1999; Leira et al., 2004; Zhao et al., 2007). Though neuroinflammation is a leading cause of neurological deficits (Yang et al., 1994; Wagner et al., 1996; Xi et al., 2006; Aronowski and Zhao, 2011; Belur et al., 2013; Zheng et al., 2016), no imaging tool is currently available to monitor brain inflammation in ICH patients. Currently, the brain inflammatory response after ICH can only be ascertained by histological examination of brain tissue sections obtained from invasive procedures like biopsy. Therefore, the development and validation of an *in vivo* biomarker of microglial activation is a major advancement to monitor brain pathology and thereby to assess the effectiveness of therapeutic interventions after ICH. To this end, we employed autoradiography studies with a second-generation TSPO ligand, [^125^ I]IodoDPA-713 as it could lay a strong platform for non-invasive neuroimaging studies after ICH.

## Can TSPO be targeted for neuroimaging after ICH?

Emerging evidences indicate a critical role of an evolutionarily well-conserved mitochondrial outer membrane protein, TSPO (18 kDa translocator protein) in neuroinflammation (Soustiel et al., 2008, 2011; Barron et al., 2013; Daugherty et al., 2013). Notably, TSPO has gained immense interest as a therapeutic target for neurologic disorders and small-molecule TSPO ligands improved functional recovery in a variety of the neurologic disorders (Soustiel et al., 2008, 2011; Barron et al., 2013; Daugherty et al., 2013). One of the key mechanisms underlying the neuroprotective effects has been highlighted as the stimulation of mitochondrial steroid synthesis with a concomitant reduction in inflammatory response (Serra et al., 1999; Verleye et al., 2005; Mitro et al., 2012; Barron et al., 2013; Zhang et al., 2014; do Rego et al., 2015). However, recent studies with transgenic mouse models demonstrate that TSPO is not essential for steroidogenesis (Banati et al., 2014; Morohaku et al., 2014; Tu et al., 2014), suggesting an elusive role of TSPO in normal physiology and neuropathology despite its augmented expression in brain inflammatory cells.

We recently demonstrated for the first time the profound induction of TSPO after ICH in comparison to sham (Bonsack et al., 2016). Further, TSPO induction after ICH was mostly confined to the peri-hematomal brain region and was mainly observed in Iba1 positive microglia/macrophage, the inflammatory cells of the central nervous system (CNS) (Bonsack et al., 2016). Notably, a profound up regulation of TSPO was observed on day 3 and day 5-post injury and the induction of TSPO after ICH mirrored the microglial activation profile after ICH (Bonsack et al., 2016). Further, the induction of TSPO paralleled and co-localized with the expression of proinflammatory and anti-inflammatory microglial markers, CD16/32 and CD206, respectively further emphasizing a possible functional role of TSPO in brain inflammatory responses after ICH (Bonsack et al., 2016). Though the precise role of TSPO in microglial/macrophage functions after brain pathology remains largely unknown, the radio labeled ligands of TSPO are widely being tested for its ability to assess brain inflammation (Callaghan et al., 2015; Damont et al., 2015; Liu et al., 2015; Loth et al., 2016; Alam et al., 2017; Crawshaw and Robertson, 2017; Fujita et al., 2017; Ishikawa et al., 2018). However, until very recently no such effort has been made after ICH. To this end, a study comprising of five ICH patients documented for the first time the feasibility of employing [^11^C] labeled first generation TSPO ligand, [^11^C]-(R)-PK11195 in monitoring microglial activation after ICH (Abid et al., 2017). However, given the small sample size of the aforementioned study (Abid et al., 2017), future work is highly warranted establishing the applicability of TSPO ligands for neuroimaging applications after ICH.

## Does [^125^ I]IodoDPA-713 confer a promising tool for tracking neuroinflammatory responses after ICH?

DPA-713 (*N*,*N*-diethyl-2-[2-(4-[methoxyphenyl)-5,7-dimethyl -pyrazolo-[1,5-α]pyrimidin-3-yl]-acetamide), a pyrazolo-pyrimidine, is a second generation TSPO ligand and less lipophilic in comparison to its previous generation counterpart, PK11195 (Endres et al., 2009). Furthermore, DPA-713 has twice the affinity for TSPO (Wang et al., 2009) in comparison to PK11195. Thereby, the use of radio labeled-DPA-713 may confer reduced non-specific binding. Consistently, PET (Positron emission tomography) imaging performed with [^11^C]DPA-713 in humans resulted in higher signal-to-noise ratio in comparison to [^11^C]-PK11195 (Doorduin et al., 2009; Endres et al., 2009). [^125^ I]IodoDPA-713, a radio ligand of TSPO, has been previously used to detect the expression of TSPO in an *in vivo* mouse model of tuberculosis and [^125^ I]IodoDPA-713 SPECT activity correlated with lung inflammation after tuberculosis (Wang et al., 2009). [^125^ I] labeled radio ligands have relatively longer half-lives (half-life of [^125^ I] is ≈ 2 months) permitting prolonged dynamic functional studies. In contrast, [^11^C] labeled radio ligands are often difficult to handle due to the short half-life of the radio nucleotide (20 min) and limited to centers having particle accelerators like, cyclotron for its synthesis. Though, a very recent study demonstrated the use of [^125^ I]IodoDPA-713 in a neuropathological condition, Sandhoff disease(Loth et al., 2016), it is largely unknown whether [^125^ I] IodoDPA-713 can be used to detect the brain expression of TSPO after ICH.

**Figure 1:**
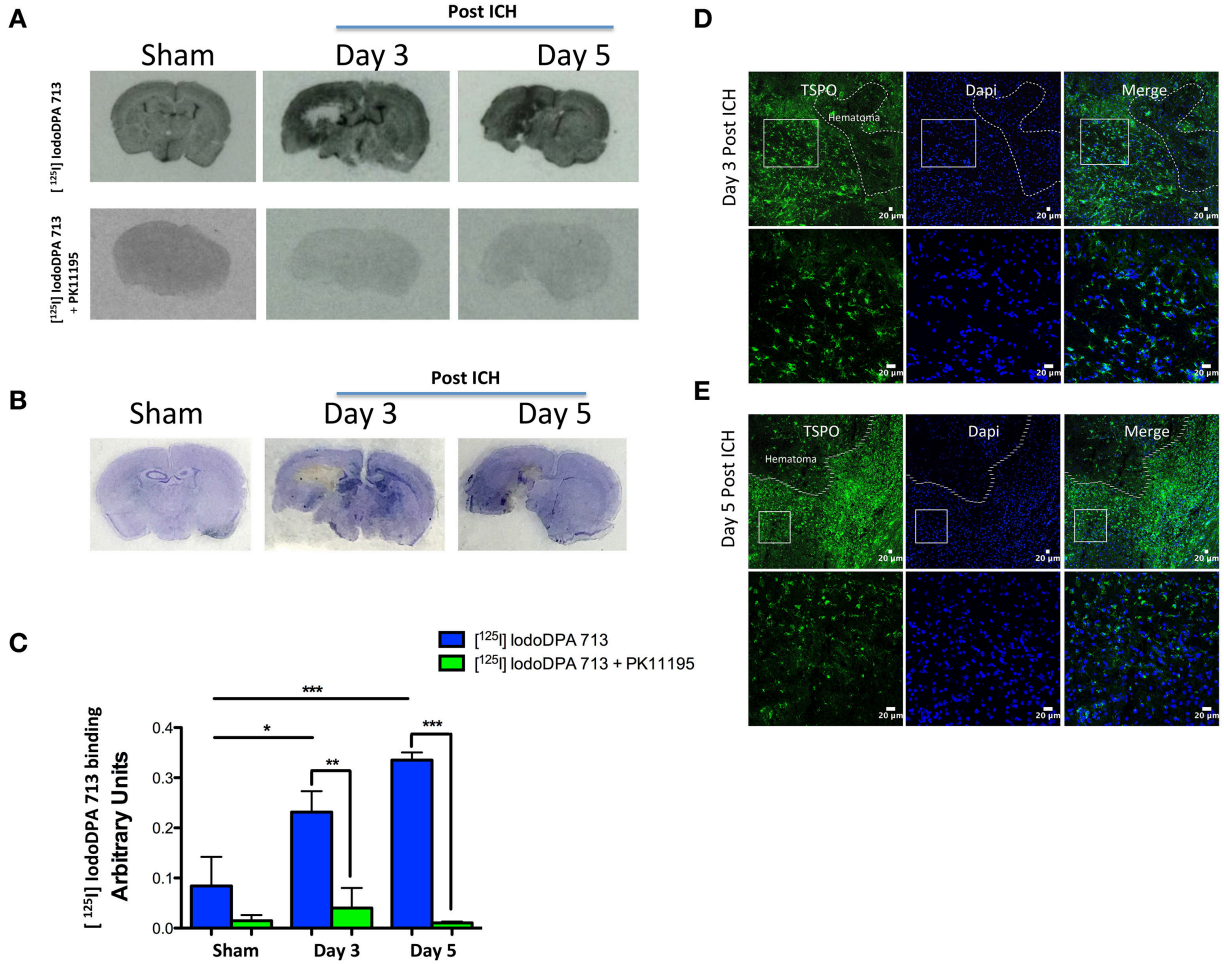
**(A)** Representative autoradiography images demonstrating the binding of [^125^ I]IodoDPA-713 to the brain sections from sham or ICH (top panel). PK11195 inhibited the binding of [^125^ I]IodoDPA-713 to the brain sections (bottom panel) (*n* = 3 mice/group). **(B)** Brain sections adjacent to the ones as depicted in **(A)** were subjected to cresyl violet staining and it demonstrates that the [^125^ I]IodoDPA-713 uptake was observed mostly in the ipsilateral striatum after ICH (*n* = 3 mice/group). **(C)** The quantification of [^125^ I]IodoDPA-713 binding to brain sections as assessed by estimating the optical density using image J (NIH, USA). ^*^p < 0.05, ^**^p < 0.01, ^***^p < 0.001 vs. control (*n* = 3 mice/group). Brain sections (*n* = 3 mice/group) were subjected to immunostaining further illustrates that the [^125^ I] IodoDPA-713 binding was observed in brain regions with enhanced TSPO expression after 3 days post-ICH **(D)** and 5 days post-ICH **(E**) and the dotted line demarcates the hematomal and peri- hematomal brain regions.

Herein, we investigate whether [^125^ I]IodoDPA-713 can be used to monitor the changes in TSPO expression in a preclinical model of intracerebral hemorrhage. To evaluate the uptake of brain sections with [^125^ I]IodoDPA-713, sham or ICH was induced in CD1 male mice as described previously (Bonsack et al., 2016) and on day 3 and day 5-post surgery, the animals were euthanized and the fresh frozen sections were used for autoradiography studies as detailed in Supplementary Methods. [^125^ I]IodoDPA-713 binding was found to be higher in the striatum after ICH in comparison to sham and the binding of [^125^ I]IodoDPA-713 was mostly confined to the peri-hematomal brain areas (Figures 1A,B). Notably, consistent with very low expression of TSPO in uninjured or intact brain (Bonsack et al., 2016), sham as well as contralateral brain areas of ICH exhibited marginal binding of [^125^ I]IodoDPA-713 (Figure 1A). Quantitative analysis further confirmed significant induction in [^125^ I]IodoDPA-713 uptake in the peri-hematomal brain areas after ICH in comparison to sham (Figure 1C). More importantly, TSPO ligand, PK11195 completely inhibited [^125^ I]IodoDPA-713 binding to brain sections suggesting a specific TSPO-dependent binding of [^125^ I]IodoDPA-713 after ICH (Figure 1A). This was further confirmed with immunohistochemistry analysis of adjacent sections, which revealed a remarkable expression of TSPO in the areas of high [^125^ I]IodoDPA-713 binding after ICH (Figures 1D,E). Notably, brain sections from post injury days, 3 and 5 exhibited maximal microglial/macrophage activation and/or TSPO expression after ICH (Bonsack et al., 2016). Further, in contrast to other brain pathologies, TSPO induction after ICH is found predominantly in Iba1 positive activated microglia/macrophages (Bonsack et al., 2016) making it an ideal molecular candidate to track microglial/macrophage associated changes after ICH.

Microglia, the resident neuroimmune cells are broadly distributed throughout the brain (Lawson et al., 1990). Microglia comprise ≈5–20% of the total glial population of the CNS and are the first non-neuronal cells to respond to a brain injury via activation (Wang and Dore, 2007; Xiong and Yang, 2015). While some microglial functions are beneficial, activated microglia also play a detrimental role after ICH (Wang and Dore, 2007; Xiong and Yang, 2015). Notably, the activated microglia are regarded as the key cellular regulators of brain inflammation after ICH based on their local release of cytokines, chemokines, prostaglandins, and reactive oxygen species (Melton et al., 2003; Nakanishi, 2003; Aronowski and Hall, 2005; Wang and Dore, 2007; Zhang et al., 2009) and the neuroinflammatory response correlates with blood brain barrier damage, cerebral edema, hematoma expansion, neurological deterioration, and poor functional outcomes (Platt et al., 1998; Hickenbottom et al., 1999; Leira et al., 2004; Zhao et al., 2007). Furthermore, neuroimmune response after ICH also regulates the brain recruitment of blood-derived monocytes/macrophages (Tessier et al., 1997; Shiratori et al., 2010; Starossom et al., 2012) and a massive infiltration of macrophages in the peri-hematomal region occurs after ICH (Min et al., 2016; Chang et al., 2017). Though the precise functional role of microglia and infiltrating macrophages after a brain injury is largely controversial, it is postulated that a key role of activated microglia and macrophages after ICH is to phagocytose the cellular debris and blood components left in the brain after hemorrhage, a process called hematoma resolution, which is vital for the functional recovery. Consistently, it is reported that brain-infiltrating macrophages after ICH are polarized to the anti-inflammatory M2 phenotype and contribute to neurological recovery after ICH (Min et al., 2016; Chang et al., 2017). Further, a human ICH study employing microarray analysis demonstrated significant up-regulation of both pro- and anti-inflammatory genes in the peri-hematomal brain region (Carmichael et al., 2008). Of note, TSPO expression is observed in both activated microglia and brain infiltrating macrophages after ICH (Li et al., 2017), and the induction of TSPO temporarily correlated with microglia activation, which persists for a long time after ICH (Wang, 2010; Yabluchanskiy et al., 2010; Bonsack et al., 2016; Li et al., 2017). In addition, our *in vitro* studies revealed a negative regulatory role of TSPO in the release of proinflammatory cytokines from murine macrophages(Bonsack et al., 2016), together making it an ideal candidate to possibly track the functional changes associated with microglia/macrophage and thereby neuroinflammatory responses after ICH. Along these lines, the autoradiography study results as outlined above provide a strong rationale for testing the applicability of [^125^ I] labeled DPA-713 for non-invasive neuroimaging studies after ICH.

## Conclusions

ICH is a fatal stroke subtype with significant public health impact. Though neuroinflammation plays a critical role in ICH pathophysiology, no imaging tool is currently available to track activation-associated microglial/macrophage changes after ICH. Along these lines, TSPO expression is observed in both activated microglia and brain infiltrating macrophages after ICH (Li et al., 2017), and the induction of TSPO temporarily correlated with microglia activation, which persists for a long time after ICH (Wang, 2010; Yabluchanskiy et al., 2010; Bonsack et al., 2016; Li et al., 2017). Given the clinical applicability of [^125^ I] labeled DPA-713 coupled with its increased binding to the peri-hematomal region in a TSPO-dependent manner, in comparison to other brain regions after ICH as demonstrated herein, future studies need to be conducted testing its potential to detect the microglial/macrophage activation after ICH. Further, given the complex pathophysiology of ICH, the applicability of [^125^ I] IodoDPA-713 for non- invasive neuroimaging (SPECT) both in the acute as well as sub acute phases of ICH needs detailed evaluation employing preclinical animal models of ICH as it would lay a strong foundation for future clinical applications.

## Ethics statement

Animal studies (protocol #2012-0459) were reviewed and approved by the Committee on Biosafety, Animal Care and Use and Radiation safety for Research and Education at Augusta University, in compliance with NIH and USDA guidelines. The protocols with regard to the synthesis of [^125^ I]IodoDPA-713 was approved by the Johns Hopkins University, Biosafety and Radiation Safety Committees.

## Author contributions

FB carried out the immunohistochemical and autoradiography studies and participated in the data analysis. MP and CF provided [^125^ I]IodoDPA-713 for the studies. CA and AA participated in timely project discussions. SS-R conceived and designed the experiments. SS-R also conducted the animal surgeries, autoradiography studies and data analysis and drafted the manuscript. All authors read and approved the final manuscript.

### Conflict of interest statement

The authors declare that the research was conducted in the absence of any commercial or financial relationships that could be construed as a potential conflict of interest.

## Abbreviations

TSPO: 18 kDa translocator protein
ICH: Intracerebral Hemorrhage
PBS: Phosphate-buffered Saline
Iba1: Ionized calcium binding adaptor molecule 1
CD 16/32: Cluster of Differentiation 16/32
Dapi: 4′,6-diamidino-2-phenylindole
SE: Standard Error
CNS: Central Nervous System
DPA-713: N,N-diethyl-2-[2-(4[methoxy-phenyl)-5,7-dimethyl- pyrazolo[1,5-a]pyrimidin-3-yl]-acetamide.
