# Supplementary material for: [125 I]IodoDPA-713 Binding to 18 kDa Translocator Protein (TSPO) in a Mouse Model of Intracerebral Hemorrhage: Implications for Neuroimaging
Source: Front Neurosci. 2018 Feb 22;12:66. doi: 10.3389/fnins.2018.00066 (PMC5826955; doi:10.3389/fnins.2018.00066)
Supplement: Supplementary file 1 [file DataSheet1.doc]

**Supplementary Methods**

The Biosafety, Animal Care and Use and Radiation Safety Committees of the Augusta University and Johns Hopkins University, approved the respective protocols in compliance with NIH and USDA guidelines.

**Induction of Intracerebral Hemorrhage:** CD-1 mice (8-10 weeks old male; Charles River) were anesthetized with ketamine and xylazine and subjected to experimental ICH (n=6) or sham (n=3), as detailed previously(Bonsack et al., 2016; Sukumari-Ramesh and Alleyne, 2016; Sukumari-Ramesh et al., 2012a, b, 2015).  The body temperature of anesthetized mouse was kept at 37±0.5°C throughout the surgical procedure using a temperature controller and mouse was positioned in a stereotaxic head frame. Then, a burr hole (0.5 mm diameter) was made 2.2 mm lateral to the bregma on the left brain hemisphere using a dental drill.  This was followed by collagenase (0.04U; Sigma, St. Louis, MO) injection with appropriate stereotaxic guidance into the brain striatum in 0.5 μL PBS using a Hamilton syringe. Sham mouse received only PBS injection and that served as experimental controls.

**Autoradiography:** Mice were anesthetized and brains were immediately snap frozen, and cut on a cryostat. Fresh frozen coronal sections (20µm) mounted onto glass slides were washed twice with 50mM Tris NaCl and incubated with 50 mM Tris NaCl containing 5 nM [^125^I]IodoDPA-713 (Wang et al., 2009)(specific activity of 1820 Ci/mmol; radiochemical purity ≈97%), or 50 mM Tris NaCl containing 5 nM [^125^I]IodoDPA-713 + 10 μM PK11195 for 30 minutes at room temperature. This was followed by washing with ice-cold 50mM Tris, and distilled H_2_O. The sections were then allowed to dry at 37°C, and apposed to Carestream Kodak BioMax MR Film (Sigma-Aldrich) for 2 hours. The autoradiograms were developed using a film developer and the binding of [^125^I]IodoDPA-713 in the peri-hematomal area was assessed by estimating the optical density (OD) using the Image J software (NIH) and the data was corrected for background signal with the OD derived from the respective contralateral brain region.

**Cresyl Violet staining.** Brain sections were fixed with 4% paraformaldehyde (5 min), treated with different dilutions of ethanol (100, 90 and 70%, respectively) and stained with 0.5% Cresyl Violet solution for 6 min. Then the sections were immersed in ethanol (70, 90, and 100%, respectively), treated with xylene, allowed to dry and subjected to imaging.

**Immunohistochemistry**

Fresh-frozen coronal sections (20μM) mounted onto glass slides were subjected to immunohistochemistry, as detailed earlier (Bonsack et al., 2016; Sukumari-Ramesh and Alleyne, 2016; Sukumari-Ramesh et al., 2012a, b, 2015). Briefly, sections were fixed with 1 % paraformaldehyde for 5 min at room temperature and washed thrice with PBS. This was followed by blocking with normal donkey serum (10%) in PBS containing Triton X-100 for 1 h and incubation with primary antibody, TSPO (1:250; rabbit monoclonal; Abcam, MA, USA) for 18h at 4^0^C. Sections were washed and treated with appropriate secondary antibody (Alexa Fluor-tagged) for 1h at room temperature. The immunofluorescence was determined using a LSM510 Meta confocal laser (Zeiss) microscope.

**Statistical analysis**

The data were analyzed using ANOVA (one-way analysis of variance) followed by Student-Newman-Keuls post hoc test and were expressed as mean ± SE. *p* value of <0.05 was considered as significant.
